# European association for endoscopic surgery (EAES) consensus statement on single-incision endoscopic surgery

**DOI:** 10.1007/s00464-019-06693-2

**Published:** 2019-02-15

**Authors:** Salvador Morales-Conde, Andrea Peeters, Yannick M. Meyer, Stavros A. Antoniou, Isaías Alarcón del Agua, Alberto Arezzo, Simone Arolfo, Amir Ben Yehuda, Luigi Boni, Elisa Cassinotti, Giovanni Dapri, Tao Yang, Sofie Fransen, Antonello Forgione, Shahab Hajibandeh, Shahin Hajibandeh, Michele Mazzola, Marco Migliore, Christof Mittermair, Doris Mittermair, Antonio Morandeira-Rivas, Carlos Moreno-Sanz, Andrea Morlacchi, Eran Nizri, Myrthe Nuijts, Jonas Raakow, Francisco M. Sánchez-Margallo, Juan A. Sánchez-Margallo, Amir Szold, Helmut Weiss, Michael Weiss, Ricardo Zorron, Nicole D. Bouvy

**Affiliations:** 1 Unit of Innovation in Minimally Invasive Sugery, Department of General and Digestive Surgery, University Hospital “Virgen del Rocio”, Sevilla, Spain; 2 Department of Clinical Epidemiology and Medical Technology Assessment, Maastricht University Medical Center, Maastricht, The Netherlands; 3 Department of Surgery, Maastricht University Medical Center, Maastricht, The Netherlands; 4 Colorectal Department, Royal Devon & Exeter NHS Foundation Trust, Exeter, UK; 5 Department of Surgical Sciences, University of Torino, Torino, Italy; 6 Surgery division, Assaf Harofe medical center, Zeriffin, Israel; 7 Department of Surgery, Fondazione IRCCS Ca’ Granda - Ospedale Maggiore Policlinico, University of Milan, Milan, Italy; 8 Saint-Pierre University Hospital, Brussels, Belgium; 9 Department of Surgery, Laurentius Ziekenhuis Roermond, Roermond, The Netherlands; 10 Niguarda Cà Granda Hospital, Milan, Italy; 11 Department of General Surgery, Royal Bolton Hospital, Bolton, UK; 12 Department of General Surgery, Stepping Hill Hospital, Stockport, UK; 13 SJOG Hospital - PMU Teaching Hospital, Salzburg, Austria; 14 Department of Surgery, “La Mancha Centro” General Hospital, Alcázar de San Juan, Ciudad Real, Spain; 15 Department of Surgery, University of Insubria, Varese, Italy; 16 Surgery division, Tel Aviv Sourasky Medical Center, Tel Aviv, Israel; 17 Center for Innovative Surgery- ZIC, Charité – Universitätsmedizin, Chirurgische Klinik, Campus Charité Mitte/ Campus Virchow-Klinikum, Berlin, Germany; 18 Jesús Usón Minimally Invasive Surgery Centre, Cáceres, Spain; 19 Assia Medical Group, Tel Aviv, Israel

**Keywords:** Single incision, Laparoscopy, Laparoscopic surgery, Consensus, Statement, Recommendation

## Abstract

**Background:**

Laparoscopic surgery changed the management of numerous surgical conditions. It was associated with many advantages over open surgery, such as decreased postoperative pain, faster recovery, shorter hospital stay and excellent cosmesis. Since two decades single-incision endoscopic surgery (SIES) was introduced to the surgical community. SIES could possibly result in even better postoperative outcomes than multi-port laparoscopic surgery, especially concerning cosmetic outcomes and pain. However, the single-incision surgical procedure is associated with quite some challenges.

**Methods:**

An expert panel of surgeons has been selected and invited to participate in the preparation of the material for a consensus meeting on the topic SIES, which was held during the EAES congress in Frankfurt, June 16, 2017. The material presented during the consensus meeting was based on evidence identified through a systematic search of literature according to a pre-specified protocol. Three main topics with respect to SIES have been identified by the panel: (1) General, (2) Organ specific, (3) New development. Within each of these topics, subcategories have been defined. Evidence was graded according to the Oxford 2011 Levels of Evidence. Recommendations were made according to the GRADE criteria.

**Results:**

In general, there is a lack of high level evidence and a lack of long-term follow-up in the field of single-incision endoscopic surgery. In selected patients, the single-incision approach seems to be safe and effective in terms of perioperative morbidity. Satisfaction with cosmesis has been established to be the main advantage of the single-incision approach. Less pain after single-incision approach compared to conventional laparoscopy seems to be considered an advantage, although it has not been consistently demonstrated across studies.

**Conclusions:**

Considering the increased direct costs (devices, instruments and operating time) of the SIES procedure and the prolonged learning curve, wider acceptance of the procedure should be supported only after demonstration of clear benefits.

Laparoscopic surgery changed the management of numerous surgical conditions. It was associated with many advantages over open surgery, such as decreased postoperative pain, faster recovery, shorter hospital stay and excellent cosmesis. Since two decades single-incision endoscopic surgery (SIES) was introduced to the surgical community. Early reports described the placement of multiple trocars through one incision with sometimes retraction of organs utilizing trans-abdominal sutures. Later, newly developed special devices were introduced to facilitate SIES.

SIES could possibly result in even better postoperative outcomes than multi-port laparoscopic surgery, especially concerning cosmetic outcomes and pain. However, the single-incision surgical procedure is associated with quite some challenges. One of the technical challenges of SIES is the loss of triangulation and therefore conflict of the instruments. Also, in obese patients a limited maneuverability of the SIES port might cause a problem. The retraction of solid organs could be sometimes problematic as well. Further, because of the use of a single entry port and thereby also a need of a larger incision size, more wound infections and incisional hernias might occur after SIES. Finally, a prolonged learning curve is a necessity to optimally execute technically demanding procedures such as SIES.

After considering all these aspects, doubts have arisen among surgeons whether SIES is a “way to go” in surgery. The main question is whether there is enough evidence to support an adoption of SIES as a safe and feasible surgical approach and consequently, if it should be routinely performed. Besides the clinical outcomes, a cost aspect should be taken into account as well. The European association for endoscopic surgery (EAES) has gathered all available evidence on this topic, and its’ members have discussed and commented on the found evidence during a consensus meeting at the EAES congress in June 2017. In this consensus paper, we try to outline the advantages and disadvantages of SIES, addressing the general aspects of this surgical procedure, as well as the organ specific issues.

The report of this consensus statement applies to general surgeons, particularly those with special interest in minimally invasive surgery, policymakers, researchers, medical device manufacturers and general practitioners, to aid in patient decision making.

## Methods

An expert panel of surgeons has been selected and invited to participate in the preparation of the material for a consensus meeting on the topic SIES, which was held during the EAES congress in Frankfurt, June 16, 2017. Two surgeons with vast experience in endoscopic surgery (NB, SM-C) and by an epidemiologist (AP) coordinated the project. The members of the panel had met three times (February, March, June 2017) before the final presentation at the EAES congress, to discuss the strategy, preparation and progress of the project.

The material presented during the consensus meeting was based on evidence identified through a systematic search of literature according to a pre-specified protocol. Three main topics with respect to SIES have been identified by the panel: (1) General, (2) Organ specific, (3) New development. Within each of these topics, subcategories have been defined. ‘General’ topics included (a) instruments, (b) devices and (c) ergonomics. ‘Organ specific’ topics included (a) cholecystectomy, (b) appendectomy, (c) colon, (d) rectum–abdominal approach, (e) bariatrics, (f) spleen and adrenal, (g) liver and pancreas, (h) upper GI-benign, (i) upper GI-malignant and (j) abdominal wall-inguinal and ventral hernia. ‘New development’ topics included (a) single-port intragastric, (b) single-port through natural orifice and (c) single-port and robotics. For each (sub) category, a member of a team has been assigned, responsible for the search, methodological appraisal of the articles, data extraction and presentation of the material.

IRB approval and written consent were not required for this paper.

### Search

The participants received a search specific to their (sub) topic. The composition of the searches has been discussed and approved by a librarian at the Maastricht University Library (The Netherlands). Searches included a query for searching the PubMed database; another query has been designed to search EMBASE (using OVID). The participants were also encouraged to search beyond the scope of the given search and consider papers for inclusion if found by an alternative way as well.

### Selection of articles

The participants have been asked to note the number of studies identified by the search, number of included and excluded studies and the reason for exclusion. They were instructed to include studies on SIES applicable to their (sub) topic. SIES procedures with and extra trocar planned from the beginning of the surgery, especially for advance procedures, were considered SIES. Inclusion criteria were: randomized controlled trials (RCTs), prospective studies, studies in patients older than 12 years. Exclusion criteria were: retrospective studies, case reports, studies in children under 12 years of age. The expectation was that for some categories it might be difficult to find randomized trials or even prospective studies. As safety is an important issue after introduction of new medical devices and operating techniques, it has been decided that in case of a serious lack of information concerning safety after the first selection, also case reports or retrospective case series with large numbers, if they added important information, could be reviewed.

### Data extraction and appraisal of the methodological quality of the studies

A uniform excel database template for entering the data extracted from the selected papers has been provided to all participants. Important outcome measures with respect to SIES have been included in the template: operating time, postoperative pain, need for additional ports, conversion to open surgery, hospital stay, postoperative pain, cosmesis, adverse events and mortality. The participants were encouraged to add any other outcome measure if necessary. The template contained predefined fields for noting important information for each study, like population characteristics, detailed information about the surgical procedure, experience level of the surgeons, etc. and the results for each outcome, including the effect size and statistical significance where appropriate. In case of malignancy, the presence of free margins, the histological specimen surrogate, the length of follow-up and tumor recurrence/disease free-survival rate were noted as well.

The methodological quality of the randomized controlled trials has been assessed with the Cochrane Collaboration’s tool for assessing risk of bias [1]. The appropriate fields to fill in the scores were included in the excel template.

### Grading of evidence/definitions

After data extraction, the teams worked out a presentation on their topic and a more comprehensive summary of their findings in a Word file. All presentations were made according to the same pre-specified format. Included in the presentations were: flowchart of the selection of articles, description of the population, summary of the papers, conclusions, statements and recommendations. The teams were asked to present preferably the results of the RCTs, potentially the results of a meta-analysis.

The teams were also specifically asked to state any adverse events, even if reported in studies of lower methodological quality. The definition of safety involved occurrence of peri- and postoperative mortality and morbidity (serious side effects).

With each “Statement” the level of evidence has been given. Grading of evidence was based on “The Oxford 2011 levels of evidence” [2], which defines five levels, ranging from Level 1 (highest evidence) to Level 5 (lowest evidence). This tool allows for grading levels down on the basis of study quality, imprecision, inconsistency between studies, etc. It also allows to grade up in case of a large or very large effect size. The participants were well informed about this. They were asked to take the outcomes of the Cochrane Collaboration’s tool for assessing risk of bias into account while grading the evidence provided by RCTs. With each ‘Recommendation’, the level of recommendation has been given. This was defined as ‘strong’ or ‘weak’ or ‘no recommendation’ according to the GRADE criteria [3].

To make sure that all teams use the same wording and phrasing, an instruction has been given on how to formulate statements and recommendations. The following wording has been proposed for the formulation of the statements: for statements based on level 1 evidence the terms ‘is’, ‘is associated’ and ‘has’ were to be used, for statements based on levels 2 to 5 of evidence the word ‘might’ was reserved. For strong recommendations, the words ‘must’ or ‘should’ had to be used and for weak recommendations the words ‘could’ or ‘might’ [4].

For the outcome ‘safety’, we have chosen also to use the term ‘seems comparable’ in the statements supported by Level 1 evidence. The reason for this is that clinical trials are usually underpowered to provide definitive results. This is to be expected, as the sample size for the clinical trials is mostly calculated on the basis of other outcomes, rather than mortality and serious adverse events.

## Results

The literature searches were performed up to February 2017.

### General topics

#### Instruments

##### Statements

1. A combination of straight and curved, or straight and articulating instruments might result in improved skills acquisition in single-incision endoscopic surgery. (LoE4)
2. A combination of two curved or two articulating instruments might be associated with worse task performance. (LoE4)

There are several types of instruments available on the market for single-port laparoscopy. These are straight, articulating, curved or double curved instruments. Sixteen studies were retrieved from the literature, in which the use of these instruments has been evaluated. These are one network meta-analysis [5], 13 RCTs [6–18] and 2 prospective studies [19, 20].

In the network meta-analysis, straight instrumentation was compared with curved or articulating instruments in the context of cholecystectomy. Significantly lower odds for addition of extra ports has been found when straight instruments were used (odds ratio (OR) 17.48, 95% confidence interval (CI) 4.03–75.74). This estimate is not very precise considering the broad interval estimates. Also in favor of the straight instruments was a shorter duration of surgery (mean difference (MD) − 32.53 min, 95% CI − 24.23 to − 40.83). The 13 randomized trials showed conflicting results but in general, a combination of straight and curved or articulating instruments resulted in improved task performance. In the two selected prospective studies, a better task performance was achieved by the use of straight over double curved and straight/curved over double curved instruments.

The above-mentioned studies have many methodological shortcomings. In 13 studies, the evaluation was done with a box trainer task assessment [6–18]; moreover, in five studies the tasks were performed by medical students [8, 9, 12, 15, 16]. In one randomized trial, in which straight instruments were compared with articulating instruments in 150 laparoscopic cholecystectomies, the external validity was limited due to untypical dissection of Calot’s triangle [14]. Another randomized trial which found that the use of straight/curved instruments lead to a better task performance than the use of double curved instruments, the laparoscopic nephrectomies were done in porcine models [6].

In general, the level of evidence provided by those studies is low. A combination of instruments including a straight component might result in better task performance, at least during the learning curve. Individual surgeons may, however, become acquainted with specific types of instruments.

##### Recommendations

1. The use of a combination of one straight and one curved/articulating instrument could be suggested during the learning curve of single-incision endoscopic surgery.

Grade of recommendation: Weak.

#### Devices

##### Statements

1. Reusable metal access devices for single-incision endoscopic surgery available nowadays might be associated with longer suturing task completion time compared to specific disposable devices. (LoE3)

**Fig. 1:**
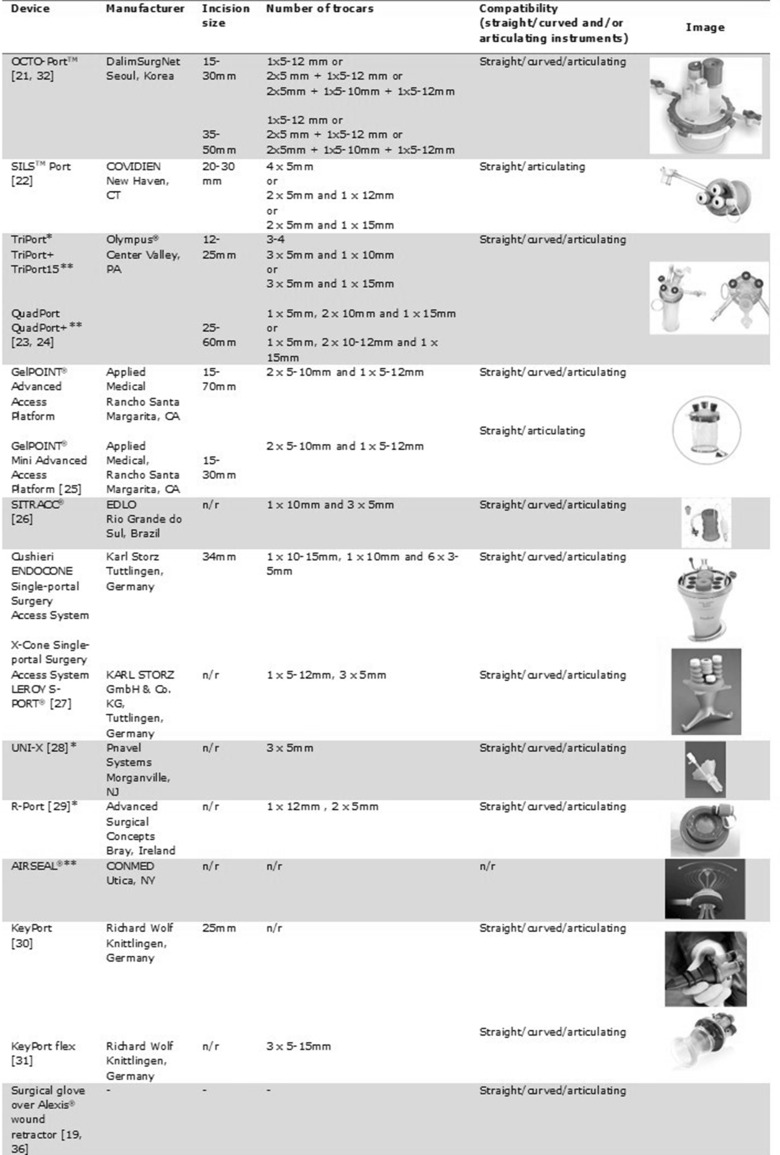
Devices. *Probably retracted. **Brochure not available on the manufacturer’s Web site;* n/r* not reported

Very limited research data are available on different devices which can be used for a single-port laparoscopic surgery (Fig. 1) [21–34]. The search yielded up one randomized trial only, which compared X-Cone, SILS™ Port and GelPOINT devices [35]. X-Cone was associated with longer suturing task completion time compared to SILS™ or GelPOINT, otherwise no differences were found. A literature search including the grey literature provided some information on 12 devices, 10 of which are available on the market. Most devices allow the use of straight, curved and articulating instruments. Two devices are reusable [36].

In the randomized trial which compared X-Cone versus SILS™ Port versus GelPOINT, 20 novices and junior surgeons participated and the assessors were blinded. Concerning the cutting task, similar completion time across groups was found. Concerning the suturing task, disposable devices were associated with shorter completion times. Further, similar performance scores across groups were found.

In conclusion, the level of evidence is very low. Disposable devices may be associated with shorter duration of specific tasks.

##### Recommendations

1. For the selection of access devices in single-incision endoscopic surgery, one could consider associated costs, taking into account that specific reusable metal devices available nowadays may be associated with longer task completion.

Grade of recommendation: Weak.

#### Ergonomics

##### Statements

1. SIES might be associated with a more neutral posture of the surgeon’s head and higher workload than conventional laparoscopic approach during video-assisted thoracic surgery. (LoE4)
2. Based on bench tests, SIES might be associated with a higher surgeon’s muscle activity and wrist’s radial/ulnar range of motion than conventional laparoscopic approach. (LoE5)
3. Based on bench tests, articulating instruments might be associated with higher surgeon’s workload, muscle activity and wrist’s radial/ulnar range of motion than straight laparoscopic instruments during SIES. (LoE5)

The literature search identified 12 studies [12, 15, 37–46] investigating the ergonomics of a single-port laparoscopic procedure. Due to a lack of larger randomized controlled trials on this topic, surveys of expert opinion and systematic reviews have also been considered. Included were nine studies, which compared SIES with conventional laparoscopy (five of which were randomized), two surveys and one systematic review. The studies are relatively small, the maximum number of subjects per study is 24, and the maximum number of participants in the surveys is 78.

In seven of these studies, a box trainer was used for the evaluation. One study used a porcine model for nephrectomy. Five studies focused on the eye-hand coordination tasks, one study on the intracorporeal suturing and one study on dissection tasks. In one study, video-assisted thoracoscopic surgery was performed. Several different types of instruments have been used, two straight instruments (four studies), one straight and one articulating instrument (three studies), two articulating instruments (three studies), two pre-bent instruments (one study). In one study, a robotic platform was involved (Single-port, multichannel surgical platform—SPSP, Spider Surgical Platform, TransEnterix). The use of the following surgical ports was reported, SILS™ port (Covidien) (two studies), TriPort™ Access System (Olympus) (one study) and GelPOINT™ System (Applied Medical) (three studies). In 2 studies, the use of three trocars through the same incision was reported.

From the 12 selected studies, nine studies were focused on comparing the ergonomics of both SIES and conventional laparoscopic instruments, two surveys evaluated the opinion of experienced surgeons regarding the ergonomic aspects of the SIES approach, and one study reviewed the ergonomic limitations imposed by SIES in comparison to multi-port laparoscopic surgery.

These are some of the main conclusions: (a) the head-trunk rotation and viewing direction are improved using SIES; (b) workload, muscle activity, and wrist’s radial/ulnar range of motion are higher with SIES than with conventional laparoscopy; (c) workload, muscle activity and wrist’s radial/ulnar range of motion are higher using articulating instruments than straight laparoscopic instruments during SIES; (d) the use of SILS™ port leads to a higher wrist’s radial/ulnar range of motion than using GelPOINT™ during SIES.

The identified evidence is of low quality, whereas some of the studies presented conflicting results. The ergonomic analyses on SIES robotic surgery are scarce. New studies with a larger number of participants, more complex tasks and performed in real conditions are required to inform on ergonomic parameters in single-port laparoscopy.

##### Recommendations

No recommendation.

### Organ specific topics

#### Cholecystectomy

##### Statements

In selected patients (elective cholecystectomy in patients with BMI < 35):

1. SIES cholecystectomy is feasible and seems safe compared to four-port laparoscopic cholecystectomy. (LoE1)
2. SIES cholecystectomy is associated with better cosmesis, lower postoperative pain and longer operative time in comparison with four-port laparoscopic cholecystectomy (LC). (LoE1)
3. In SIES cholecystectomy, length of hospital stay and quality of life are comparable to four-port laparoscopic cholecystectomy. (LoE1)

SIES cholecystectomy is a new concept in minimally invasive surgery. The access to the operative field is gained through the natural scar of the umbilicus, which makes this procedure even less invasive than the standard laparoscopic approach. Surgeons all over the world are still exploring the concept of single-port surgery. SIES cholecystectomy is the most frequently performed procedure with a single-port approach to date.

11 RCTs were selected for review [47–57]. There are pooled data available of the results of nine of the selected RCTs (n = 860) as these were recently included in a systematic review with a meta-analysis [58]. Also, recently the results of a large international multi-center non-inferiority trial (n = 600) were published [56]. Additionally, a smaller randomized trial (n = 64) was found [57].

All RCTs compared SIES cholecystectomy with conventional four-port laparoscopic cholecystectomy (LC). The total population consists of 1524 adult patients, and mean age is comparable across the studies and also for the SIES and LC group, ranging from 46 to 50 years. All patients have ASA I-III grades. Most of the studies included only patients with BMI < 30 kg/m^2^ or < 35 kg/m^2^. One small study (n = 34) used a BMI < 40 kg/m^2^ as an inclusion threshold. Previous abdominal surgery and the presence of acute cholecystitis were exclusion criteria in all studies. The follow-up period ranged from 1 to 12 months, only one study (n = 60) had a longer follow-up, up to 16 months.

Compared to laparoscopic cholecystectomy, cosmesis was significantly better after a SIES procedure. This was according to the meta-analysis and also according to the results of the non-inferiority trial, although in this trial the surgeons rated the cosmetic results of the conventional LC group as better compared to patients self-assessment. The smaller randomized study found no difference in cosmetic results. This study and the pooled data of the nine RCTs showed significantly lower scores of postoperative pain for SIES. In the non-inferiority trial, no difference was found. All results suggested a significantly longer operating time for the SIES procedure, with mean difference ranging from 15 to 30 min. The length of hospital stay and quality of life were comparable for both procedures. Both, the meta-analysis and the non-inferiority trial, found no difference for these outcomes.

Mortality did not occur in any of the studies. According to the meta-analysis of the nine RCTs, there was a higher risk of severe adverse events in the SIES group than in the LC group. In these pooled data, serious adverse events included bile duct injury, re-operations, intra-abdominal collections or bile leaks requiring drainage or infected intra-abdominal collections. However, a bile duct injury occurred in two patients only, in one patient after a SIES procedure and in one patient after a LC procedure. In the large non-inferiority trial and the smaller randomized trial, no difference in severe adverse events was found (no bile duct injuries occurred). The meta-analysis data showed a higher risk of mild adverse events in the SIES group but statistical significance was not reached. In the non-inferiority trial, no difference was found.

There was a significantly higher need of additional ports in the SIES group according to the meta-analysis, but this was actually due to the results of one of the nine pooled studies, in which 17 of 60 patients needed an extra umbilical access during SIES. In the non-inferiority trial, no difference in the use of additional ports was found. No difference was found in conversion to open cholecystectomy. There were seven port-site hernias reported in the SIES group and one in the LC group out of 430 patients in each arm, in the nine trials included in the meta-analysis. In the non-inferiority trial, the absolute numbers were six and three, respectively.

The meta-analysis of the nine trials and the large non-inferiority trial provided no evidence on costs. The smaller randomized trial suggested earlier return to work and less need for use of analgesics in the SIES group, whereas the costs of the SIES procedure were higher. In the non-inferiority trial, approximately equal amount of analgesics was used in SIES and LC groups.

Overall, evidence on SIES cholecystectomy suggests better cosmetic results and less postoperative pain when compared to standard LC. On the other hand, the operating time is longer. Concerning morbidity, discrepant results were found. The pooled data of nine trials showed a higher risk of adverse events after the SIES procedure, whereas the non-inferiority trial showed that SIES was non-inferior to LC in terms of safety. This non-inferiority trial was primarily designed to prove non-inferiority of the SIES procedure compared to LC regarding morbidity within 60 days after surgery. The sample size calculation was based on this, considering a clinically significant difference of 4%. It was conducted in six countries and included 600 patients. It is the largest trial in which the outcomes of patients were compared who were enrolled according to exactly the same inclusion/exclusion criteria and who were treated under same conditions in comparable circumstances. Although in a meta-analysis the data are being pooled only in case of clinical and statistical homogeneity between the studies, still there might be differences, which can influence the outcomes. One important issue to mention is the surgical experience of the participating surgeons. In the non-inferiority trial, the first operator performed at least 50 cholecystectomies and had experience in at least 15 cases with SIES cholecystectomy. From the nine trials included in the meta-analysis, in five of them the surgeons had previous experience in SIES, three mention only experience in laparoscopic surgery, and one trial gives no information on this topic. In one RCT, the difference in the experience level of surgeons is mentioned as a study limitation. Another point to mention is the patients’ BMI. In the non-inferiority trial, only patients with BMI lower than 30 were included. Some other trials included also patients with BMI until 35 (or even 40).

No statement can be made regarding the difference in occurrence of common bile duct lesions since overall incidence of common bile duct lesions is very low. No statement can be made on risk of port-site hernia due to short follow-up.

Regarding costs of the SIES procedure, very little evidence was found.

##### Recommendations

1. SIES cholecystectomy could be performed if a patient is looking for better cosmesis and less pain compared to conventional four-port laparoscopic cholecystectomy in patients with a BMI < 35.

Grade of recommendation: Weak.

#### Appendectomy

##### Statements

In selected patients (non-perforated appendicitis):

1. SIES appendectomy is feasible and seems to be safe compared to standard laparoscopy. (LoE1)
2. SIES appendectomy is associated with better cosmetic outcomes, shorter hospital stay and earlier return to work compared to standard laparoscopy. (LoE1)
3. SIES appendectomy is associated with outcomes comparable to standard laparoscopy with regard to operating time and postoperative pain. (LoE1)

Over the past years laparoscopic appendectomy (LA) has become the treatment of choice for acute appendicitis. The laparoscopic procedure is associated with less postoperative pain, lower postoperative complication rates and earlier recovery, compared to open appendectomy. Single-incision endoscopic surgery in which the number of ports is reduced seems to be suitable for appendectomy in non-perforated appendicitis. To assess the presumed benefits and safety of this procedure, several studies have already been conducted in which SIES appendectomy (SIEA) is compared to conventional laparoscopic appendectomy.

12 RCTs in which patients were randomized to receive either SIEA or conventional LA were eligible for the review [59–70]. Trials in which extra sutures, trocars or K-wires were used were excluded. The results of the 12 studies were pooled in a meta-analysis for several outcomes, when the data were suitable for pooling. Members of the consensus team who were responsible for this topic have conducted the meta-analysis according to the guidelines from the PRISMA statement for reporting systematic reviews and meta-analyses, with a purpose to publish these results in detail as a separate publication (yet to be published).

The total study population consists of 1524 patients, 759 patients underwent SIEA and 765 patients underwent conventional LA. The mean age is 30,6 years and 32,4 years, respectively. The ASA grades were reported in three studies only (n = 247), and most patients have a score ASA I. The mean BMI is 23,1 kg/m^2^ (SIEA) and 23,6 kg/m^2^ (LA). The surgical experience was inconsistently reported. Two trials reported that the surgeons had performed more than 25 SIEA (n = 75) and more than 20 SIEA (n = 195) procedures, respectively, in the past. Two studies (n = 271) reported that all participating surgeons were experienced, but did not further quantify the experience. In one trial (n = 77), it was stated that more experienced surgeons were operating on SIEA patients. The follow-up duration varied considerably between the studies, ranging from 14 days to 24 months. Only two out of twelve studies had a follow-up duration longer than 6 months, namely 20,8 months (n = 102) and 24 months (n = 120).

The pooled data (five studies, n = 484) showed significantly better postoperative cosmetic scores in the SIEA group compared to LA group. There was no difference in postoperative pain at 12, 24 and 48 h postoperatively (eight studies, n = 1411). The operating time (11 studies, n = 1437) was similar in both groups, with an average of 52 min in the SIEA group and 49 min in the LA group. Hospital stay (11 studies, n = 1295) was significantly shorter in the SIEA group, with a mean difference of 0,11 days (27,4 min). Patients also returned earlier to work after the SIEA procedure (four studies, n = 449), the mean difference was 0,61 days.

No mortality was reported in any of the studies. The results of all 12 studies (n = 1524) were pooled for the outcome ‘adverse events’. There was no difference in the occurrence of serious adverse events (SAEs) or mild adverse events (MAEs) between the groups. In the SIEA group, there were 14 SAEs compared to 13 SAEs in the LA group. The occurrence of MAEs was 59 in the SIEA group and 68 in the LA group. This difference was no statistically significant. Three patients developed a port-site hernia (seven studies, n = 702); all three were in the SIEA group.

There was no difference in conversion rates to open appendectomy. In the SIEA group, significantly more additional ports were used compared to the LA group, namely 22 to 2, respectively (seven studies, n = 801). One study found significantly higher costs for the single-port procedure compared to conventional LA (n = 120) [70]. No further information on costs could be retrieved.

In conclusion, patients who underwent single-port appendectomy were more satisfied with the cosmetic outcomes than patients who underwent a conventional laparoscopic approach. There was, however, no difference in postoperative pain. The hospital stay and return to work were both in favor of the single-port approach, but the profit is relatively small when expressed in absolute figures. The single-port approach required more use of additional ports. There was no difference in adverse events.

The data on port-site hernias are limited due to a short follow-up in most studies. No statement is formulated on this outcome. Cost comparison could not be made.

##### Recommendations

1. SIES appendectomy in non-perforated appendicitis could be performed if patient is looking for better cosmesis and earlier return to work.

Grade of recommendation: Weak.

#### Colon

##### Statements

In selected patients (< T4 or tumors < 5 cm, BMI < 35, no previous abdominal surgery):

1. SIES colectomy might be safe and feasible. (LoE2)
2. SIES colectomy might be associated with same oncological surrogate outcome as multi-port laparoscopic colectomy, but long-term data on oncological outcomes are lacking. (LoE2)
3. SIES colectomy might have comparable perioperative outcomes as multi-port laparoscopic colectomy regarding morbidity and complication rate. (LoE2)

Since the first reported laparoscopic colectomy in 1991, the safety of the laparoscopic approach in colorectal surgery and equivalent or even better oncologic outcomes in colorectal cancer have been demonstrated. The advantages of laparoscopic colectomy over open surgery include shorter hospital stay and faster recovery of bowel function, reduced blood loss, less postoperative pain and better cosmesis. SIES could even maximize the specific benefits of laparoscopic approach, especially regarding incisional trauma, postoperative pain and wound related complications.

Three RCTs were found in the literature in which single-port (SP) colectomy was compared to multi-port (MP) colectomy in patients with colorectal neoplasms [71–73]. The number of patients included in the randomized trials is relatively low. Therefore, also prospective and retrospective studies (with prospective data collection) conducted on large cohorts of patients were reviewed for complications and other relevant outcomes [74–102].

One RCT included 200 patients, 100 in each treatment arm, whereas the other two RCTs were rather small, with 50 and 16 patients. Surgeries included right and left colectomies, sigmoid resections and low anterior resections. Included were patients with tumors < T4 or tumors < 5 cm, BMI < 35 kg/m^2^, without previous abdominal surgery. Patients with previous history of peritonitis, ASA score 4 and emergency surgery were excluded. Overall, no differences were found for perioperative and short-term postoperative outcomes. The mean number of resected lymph nodes was comparable between the SP and MP groups. There were no deaths, and the complication rates were similar. In one trial (n = 50), the patients in the SP group had significantly lower median pain score on day 1 and day 2. The median hospital stay in the SP group in this trial was shorter than in the MP group.

The randomized trials focused on short-term outcomes after surgery. The reported oncological outcomes are “surrogate” outcomes, such as number of lymph nodes harvested, free margins and length of the specimen. The largest trial (n = 200) is still on-going [72]; the disease-free survival 5 years after surgery will be reported after termination of the follow-up.

The results from the other included studies support the findings of the randomized trials. In terms of safety, all studies indicate that single-incision approach in colonic surgery is a safe and feasible. The oncological outcomes, whether surrogate oncological outcomes or recurrence rates or disease-free survival, all show results comparable to standard laparoscopy for cancer. Operative times were inconsistent across the studies. This might be associated with selection of patients for SP surgery and also by the experience of surgeons with this technique. Some studies reported on faster postoperative recovery and shorter hospital stay after SP. Postoperative pain-control seemed to be also more favorable for the SP group. The complication rates did not seem to differ between the SP and MP approaches. The cosmetic results were not specifically reported in the studies.

##### Recommendations

1. In selected patients (< T4 or tumors < 5 cm, BMI < 35, no previous abdominal surgery) SIES colonic resection could be offered to patients as an equally safe and effective alternative compared to multi-port colonic surgery with comparable histological surrogate outcome.

Grade of recommendation: Weak.

#### Rectum

##### Statements

In selected patients (tumor < 4 cm, BMI < 30):

1. SIES rectum resection might be a safe procedure with comparable outcomes as multi-port laparoscopy, if carried out by experienced surgeons. (LoE2)
2. The postoperative pain might be lower after SIES rectum resection compared to multi-port laparoscopy. (LoE2)
3. The histological surrogate outcome for malignant indications might be comparable between SIES rectum resection and multi-port laparoscopy. (LoE2)

Laparoscopic surgery for rectal disease has been proven to be equivalent to open surgery in randomized studies in the last years. Nonetheless, the adoption of SIES rectal surgery by surgeons is still low, possibly due to the complexity of the procedures and an extended learning curve. However, as a large extraction site or planned diversion stoma site is needed in many cases for colorectal resections, there can be a rationale for the use the single access for rectal resections.

One RCT [103] (n = 40) and six comparative studies [104–109] (n = 670), in which SIES and multi-port rectal surgery were compared were retrieved from the literature. Three of the six comparative studies started the SIES rectal resection already with one extra port. All studies included both, low anterior and abdomino-perineal resections. In most of the studies, the indication was malignant rectal cancer. Two studies also included benign rectal disease, besides the rectal cancer. Included were mainly patients with a BMI < 30 kg/m^2^ and tumors sized less than 4 cm.

The RCT was a small pilot study in which patients with rectal cancer were randomized either to SIES (n = 20) or multi-port laparoscopic surgery (n = 20). Patients after SIES rectal resection had significantly less postoperative pain during the first 4 days than patients undergoing a multi-port laparoscopic rectal resection. The incision length was significantly shorter in the SIES group. All other outcomes were similar between the groups, including operating time, blood loss, morbidity and mortality. The short-term oncological outcomes were also comparable.

The other studies reported on similar outcomes to those of the RCT. The clinical postoperative outcomes and the short-term oncological outcomes were similar between the groups. In a relatively large study (n = 55 SIES; n = 327 conventional laparoscopy), SIES was associated with shorter operating time, less postoperative pain, faster recovery and earlier discharge. Shorter hospital stay was reported in one other study as well (n = 100). One study reported shorter abdominal incisions, less pain and better satisfaction with cosmetic results after SIES (n = 57). One study estimated also the costs; these were similar for both procedures.

##### Recommendations

1. Single-incision endoscopic rectal surgery in selected patients (tumor size < 4 cm and BMI < 30) could be performed by experienced laparoscopic surgeons safely offering less postoperative pain and comparable histological surrogate outcome in comparison with multi-port laparoscopy.

Grade of recommendation: Weak.

#### Bariatrics

##### Statements

In selected patients (BMI < 50, no previous surgery, xipho-umbilical distance less than 25 cm):

1. SIES bariatric surgery might be as safe as the conventional laparoscopic approach when performed by skilled surgeons, with comparable weight loss results in short-term follow up. (LoE3)
2. SIES sleeve gastrectomy, compared to the conventional laparoscopic procedure, might be associated with less postoperative pain and a better cosmetic result, but with an increase in operative time. (LoE2)
3. SIES gastric bypass, compared to the conventional laparoscopic procedure, might be associated with less postoperative pain and a better cosmetic result, but with an increase in operative time. (LoE3)

Laparoscopic approach is nowadays the gold standard in surgical treatment of morbid obesity. Minimally invasive surgery has been proven beneficial regarding postoperative morbidity and mortality in bariatric surgery [110]. As SIES procedure might reduce the level of postoperative pain, it is being explored as a surgical option in patients with morbid obesity, despite the fact that other SIES procedures are preferably not being performed in patients with a BMI > 35 kg/m^2^. In case of sleeve gastrectomy, a larger incision is used anyway to extract the remaining stomach tissue so one can reduce the number of further incisions in the abdominal wall using the single-incision approach. In case of gastric bypass, it seems, however, more complicated to use SIES because of the requirement for intracorporeal sutures and anastomoses.

The literature search identified 10 studies in which patients underwent a SIES procedure for the treatment of morbid obesity. All are comparative studies, evaluating SIES versus multi-port conventional laparoscopy (CL). In eight studies, the performed procedure is sleeve gastrectomy [111–118] and in two studies gastric bypass [119, 120]. Liver retraction was achieved by either liver suspension tape attached to two Prolene sutures or a 3-mm mini liver retractor. Furthermore, one systematic review published in 2015 was identified [121]. However, we were not able to retrieve any pooled data as no meta-analysis was undertaken. The authors included also studies in which laparoscopic gastric banding was used. As this procedure is being used rarely nowadays, studies on SIES laparoscopic gastric banding were not evaluated in our analysis. Only one of the studies from our literature search is an RCT [111], the other studies are three prospective and six retrospective comparative studies. The prospective studies were reviewed for patient-related outcomes, and the retrospective studies were reviewed for major complications.

The RCT is a pilot study in which 30 patients were randomized either to SIES (n = 15) or CL (n = 15) sleeve gastrectomy. Included were patients with BMI < 50 kg/m^2^ and xipho-umbilical distance less than 25 cm. All patients are female except for two males in the SIES group. The mean BMI is 44,35 kg/m^2^ and 45,52 kg/m^2^ in the SIES and CL groups, respectively, the mean ASA score is 2,41 in the SIES and 2,33 in the CL group. There were no intra- or postoperative complications in either group; all patients were discharged on the third day post-surgery with instructions for a liquid diet. No differences were found regarding pain at rest, operative time or weight loss at 6 months. Patients in the SIES group reported significantly less pain during movement on the first and second day postoperatively compared to the patients in the CL group, but on the third day no significant difference in pain scores was found. The SIES patients reported a significantly higher aesthetical satisfaction at 1, 3 and 6 months.

In two prospective studies with sleeve gastrectomy, with even number of patients undergoing SIES and CL, single-incision technique was found to be technically feasible with results that were mostly similar to those obtained with multi-port conventional laparoscopy. In the larger study (n = 600), the SIES procedure was scored as less painful with better cosmesis. Three patients in the SIES group developed an incisional hernia compared to no patients in the CL group within a follow-up of up to 2 years. In the smaller study (n = 42), no differences in morbidity or hospital stay were found between the groups. Operative time was higher in the SIES group. There was one conversion to laparoscopic surgery in the single-incision group. In the retrospective studies with gastric sleeve surgery (n = 147 SIES), no major complications or occurrence of incisional hernias were reported. The longest follow-up was 1 year.

Only one of the two studies on SIES gastric bypass surgery was performed prospectively (n = 40 SIES, n = 100 CL). No difference in complications compared to multi-port laparoscopy was found. The operative time was longer in the SIES group. The recovery and weight loss were comparable between the groups. The SIES procedure resulted in better patient satisfaction. The follow-up was up to 12 months. In the retrospective study with gastric bypass surgery (n = 100 SIES, n = 100 CL), 18 patients required an extra skin incision for a 5 mm port. Complications were equally distributed in the two groups.

No mortality occurred in any of the reviewed studies.

##### Recommendations

1. In a controlled environment of expert bariatric surgeons, single-incision laparoscopic bariatric surgery (sleeve gastrectomy and gastric bypass) could be performed safely in selected patients (BMI < 50, no previous surgery and xipho-umbilical distance less than 25 cm), especially in those concerned about cosmetic results.

Grade of recommendation: Weak.

#### Spleen and adrenal

##### Splenectomy

*Statements*

In selected patients (estimated spleen weight ≤ 500 g):

1. SIES splenectomy might be considered a safe and feasible surgical approach with perioperative morbidity comparable to standard laparoscopic splenectomy. (LoE4)
2. SIES splenectomy might be considered superior to standard laparoscopic splenectomy in terms of cosmesis. (LoE4)
3. SIES splenectomy might be considered to require longer operative time compared to standard laparoscopic splenectomy. (LoE4)

Theoretically, the single-incision technique might be less suitable for splenectomy than for other surgical indications. A practical limitation might be the poor exposure of the lesser sac and the upper pole of spleen. Furthermore, in patients with high body mass index (BMI) or in tall patients, the surgeon might experience difficulty in reaching the spleen.

After the literature search two prospective [122, 123] and four retrospective comparative studies [124–127] have been selected for review, in which SIES splenectomy is compared to standard (or reduced port) laparoscopic splenectomy (LS). No randomized clinical trials have been published on this topic, which may be related to the fact that elective splenectomy is a rarely performed operation. As the number of patients included in the prospective studies is relatively small, the results of retrospective studies have been taken into account to document the incidence of complications.

One prospective study which compared SIES (n = 8) and standard LS (n = 15), included patients with spleen weight less than 500 g [122]. There was no conversion to open surgery in any group. Operative time was longer for surgery with the SIES technique. There were no differences in intra- or postoperative outcomes. Patients in the SIES group were more satisfied with the cosmetic results.

Another prospective study compared SIES (n = 19) and three port LS (n = 21) [123]. Operative time was longer in the SIES group. One patient in the SIES group had conversion to laparotomy due to bleeding. Postoperative pain was less in patients after SIES. In both groups, one pancreatic fistula occurred.

In the retrospective studies (n = 72 SIES and n = 85 LS), no hernia formation was reported during the follow-up period up to maximum 34 months. There was one report of intraoperative gastric wall injury in the SIES group. Bleeding rates seemed equally distributed in both techniques.

##### Recommendations

No recommendation.

##### Adrenalectomy

*Statements*

1. SIES transabdominal adrenalectomy might be considered a feasible surgical approach. (LoE4)
2. SIES transabdominal adrenalectomy might be considered similar to standard laparoscopic adrenalectomy in terms of perioperative and postoperative outcomes. (LoE4)

Multi-port laparoscopic adrenalectomy is a well-established procedure and can be performed trans- or retroperitoneally for both the left and the right adrenal. SIES for adrenalectomy may be associated with better cosmetic results.

Review of the literature identified one prospective comparative study [128], in which SIES left transperitoneal adrenalectomy was compared to multi-port laparoscopic left transperitoneal adrenalectomy (LA). Also one systematic review and meta-analysis of retrospective studies has been identified, which was published in 2016 [129].

In the selected prospective study, there were 40 patients in each treatment group. None of the SIES patients required conversion to an open procedure. In one case, an additional 5-mm port was needed for kidney retraction. No differences in operative time, nor postoperative complications were reported. Pain scores were comparable in both groups. Cosmesis was not evaluated.

In the systematic review and meta-analysis, 10 retrospective studies (n = 704) were analyzed. The sample size of the studies ranged from 9 to 140 patients. Patients in the SIES group (n = 255) had a shorter hospital stay and lower postoperative pain scores than patients in the LA group (n = 449). No differences were found in operative time, doses of required analgesics, perioperative complications and conversion rates.

##### Recommendations

No recommendation.

#### Liver and pancreas

##### Liver

*Statements*

In selected patients (various indications):

1. SIES liver resection might be performed safely and with comparable outcomes to multi-port laparoscopic resection if carried out by experienced surgeons. (LoE3)
2. SIES liver resection might result in shorter postoperative hospital stay compared to multi-port laparoscopy. (LoE3)

Laparoscopic liver surgery has not been adopted by liver surgeons as much as other laparoscopic operations. Types of laparoscopic liver surgery vary in complexity and difficulty. The most frequent indication for the laparoscopic single-incision technique is benign left-lateral liver diseases, as these are technically less demanding. The potential advantage of single-incision approach might be earlier recovery and reduced scar formation.

One RCT was identified through the literature search, in which single-port (SP) and multi-port (MP) laparoscopic left lateral liver sectionectomies (LLLS) have been compared [130]. Another four comparative studies comparing SP and MP laparoscopic liver surgery were identified [131–134].

In the RCT, all patients were operated on for benign liver diseases and they were assigned either to single-port (n = 19) or multi-port (n = 19) laparoscopic LLLS. Mean operative times did not differ significantly between groups (105 ± 23 min vs. 90 ± 27 min for SP versus MP, respectively). Postoperative hospital stay was significantly shorter in the single-port group (2.5 ± 1.7 days vs. 4.0 ± 2.1 days for SP vs. MP, p < 0.05, respectively). No conversion to open surgery was reported, whereas one conversion to multi-port laparoscopy was reported in the SP group. No deaths or clinically relevant complications were reported.

In the four comparative studies, a total of 194 patients underwent either SP (n = 107) or MP (n = 87) minor liver resections. The surgical procedures were: unroofing of liver cysts, left lateral sectionectomies, common bile duct explorations and laparoscopic part of donor right hepatectomies. Experienced surgeons performed the procedures; however, no clear definition of the grade of experience was provided. No clear procedural advantages could be established. Two studies reported on reduced operative time for SP, whereas no differences were found in other reports. Pain, cosmesis, incidence of port-site hernia and other long-term complications were inconsistently reported.

##### Recommendations

1. SIES minor liver resections could be offered as safe and effective surgery when performed by experienced surgeons compared to conventional laparoscopic approach.

Grade of recommendation: Weak.

#### Pancreas

##### Statements

In selected patients (distal pancreatic resection):

1. SIES pancreatic resection might be performed safely and with comparable outcomes as with multi-port laparoscopy, if carried out by experienced surgeons. (LoE3)
2. Operating time might be longer for SIES pancreatic resection compared to multi-port laparoscopy. (LoE3)

Laparoscopic distal pancreatectomy is increasingly being performed worldwide, in specialized surgical institutions. Although in a limited extent until now, next to a multi-port (MP) laparoscopy also a single-port (SP) laparoscopic approach in pancreatic surgery is being explored.

There are no RCTs available in the literature. Four comparative studies reporting on a total of 119 patients (n = 50 SP and n = 69 MP) were retrieved [135–138].

All studies reported on distal pancreatectomy for both, benign and malignant disease. Experienced surgeons performed the surgeries, but no definition of the grade of experience was reported. Three studies compared the SP technique with a standard four-port left sided pancreatectomy [135–137]. One study compared the SP technique with a robotic MP approach [138]. The operative strategy was comparable between the different groups and centers. The parenchyma dissection, as a critical step of the resection, was carried out by means of an endoscopic linear stapler in all patients.

In one study (n = 12 SP, n = 28 MP), the mean operative time was documented to be longer in the SP group (280 ± 53 min vs. 187 ± 87 min). The same article reported on shorter mean hospital stay after MP laparoscopic distal pancreatectomy (12.2 ± 5.4 days vs. 8.3 ± 4.7 days). This was due to a higher proportion of patients with pancreatic fistulas in the SP group. These patients namely had a longer hospital stay compared to patients without fistulas (14.7 ± 7.7 days vs. 7.9 ± 3.0 days). All other procedural outcome parameters did not differ between the two groups. Only three pancreatic fistulas (2.5%) were reported. There is no sufficient data to allow assessment of postoperative pain, cosmesis, hernia and procedural costs.

##### Recommendations

1. SIES distal pancreatic resections could be offered as an equally safe and effective procedure compared to multi-port laparoscopy, when performed by experienced surgeons.

Grade of recommendation: Weak.

#### Upper gastrointestinal tract-benign

##### Statements

In selected patients (Nissen fundoplication, ASA 1 and 2):

1. SIES Nissen fundoplication might be considered a safe and effective procedure in the short term. (LoE3)
2. SIES Nissen fundoplication might have better cosmetic results compared to standard laparoscopy. (LoE3)
3. SIES Nissen fundoplication might be associated with longer operative time compared to standard laparoscopy. (LoE3)
4. SIES Nissen fundoplication might require hospital stay comparable to standard laparoscopy. (LoE3)

Laparoscopic fundoplication has become the standard surgical treatment of gastroesophageal reflux. It has several advantages when compared to open fundoplication, such as less postoperative pain, decreased mortality and shorter hospital stay. SIES might also lead to better cosmetic results due to a single-port entry instead of multiple entries. This single-port technique is being explored now for utilization in laparoscopic antireflux surgery.

Two studies were identified in the literature, which compared the outcomes of single-site Nissen fundoplication with the conventional multi-port laparoscopic fundoplication [139, 140]. Both studies are reports, one prospective (n = 260) and one retrospective (n = 33). No RCTs have been published yet.

Both studies reported on patients with comparable age, sex and BMI distribution. ASA grades were low, mostly grade 2. The mean BMI varied from 27 to 29 kg/m^2^. The mean follow-up was 26–28 weeks. The prospective study analyzed the postoperative outcomes of consecutive patients, 130 patients underwent SIES fundoplication and 130 conventional laparoscopic fundoplication (CL). Symptom resolution was comparable between the groups. Cosmesis scores were superior for the SIES procedure, and 96% of patients were very satisfied with the cosmetic outcomes after SIES. Operative time was longer for the SIES procedure, 146 min (median) versus 101 min (median). Duration of hospital stay was comparable. There were no conversions to open surgery in the SIES group; there were seven conversions in the CL group. Further, no notable complications occurred.

In the retrospective report, the outcomes of 15 SIES and 18 laparoscopic Nissen fundoplications were compared. All patients in the SIES group achieved symptomatic relief of gastroeshophageal reflux. Mean operative time was longer in the SIES group, namely 182 min (range 111–273) versus 129 min (range 101–184). Duration of hospital stay was comparable. There were no conversions to open surgery in the CL group, whereas six of the 15 patients in the SIES group required insertion of two to four additional ports. There was no perioperative mortality or morbidity in either group. A single surgeon who had no previous experience in SIES Nissen fundoplication performed all the procedures in this study.

In conclusion, according to these reports SIES Nissen fundoplication seems to be safe and feasible, with similar symptom improvement and superior cosmesis when compared to multi-port laparoscopic procedure. No conclusions could be drawn with regard to postoperative pain and port-site hernia occurrence after SIES fundoplication. The follow-up in both reports was relatively short. For a sound comparison, randomized controlled trials with a longer follow-up time are necessary. One of the challenges for the optimal performance of this technique might be a longer learning curve.

##### Recommendations

1. SIES antireflux surgery (Nissen fundoplication) could be offered as a procedure being performed safely in selected patients (ASA 1 or 2).

Grade of recommendation: Weak.

#### Upper gastrointestinal tract malignant

##### Statements

In selected patients (early distal gastric cancer - stage Ia/Ib, BMI < 25):

1. SIES gastrectomy might be as safe in terms of postoperative complications as multi-port laparoscopic gastrectomy. (LoE4)
2. SIES gastrectomy might be associated with shorter hospital stay than multi-port laparoscopic gastrectomy. (LoE4)
3. SIES gastrectomy might have similar oncological surrogate outcomes as multi-port laparoscopic gastrectomy. (LoE4)

Single-incision laparoscopic surgery has been explored, among other indications as an option for performing gastrectomy in patients with gastric cancer.

No randomized trials have been found in the literature on this topic. However, some studies addressed the feasibility and safety of single-port gastrectomy (SIESG). Three comparative studies were found in which laparoscopic single-port distal gastrectomy for early gastric cancer was compared to multi-port (or reduced port) laparoscopic gastrectomy (MLDG) [141–143]. All studies reported on early gastric cancer, stage Ia/Ib, in patients with BMI < 25 kg/m^2^. These studies included a total of 188 patients in the SIESG group and 181 in the MLDG group. These were retrospective studies of prospectively collected data on SIESG. The data were matched with the data of patients who underwent MLDG. There was no difference in number of harvested lymph nodes. Operative time was comparable. One study reported shorter hospital stay and all studies reported faster recovery, including less pain and earlier oral intake after SIESG. Postoperative complications after 30 days were comparable; only one study (n = 90 SIESG, n = 85 MLDG) reported on survival, the 5 years overall survival rates were comparable between SIESG and MLDG [143]. There were no differences in incidence of incisional hernia in this study.

##### Recommendations

No recommendation.

#### Abdominal wall

##### Inguinal hernia

*Statements*

In selected patients (elective primary unilateral and bilateral inguinal hernia):

1. SIES TEP is feasible and seems a safe procedure when performed by experienced surgeons. (LoE1)
2. SIES TEP might require longer operative time than conventional TEP. (LoE2)
3. The postoperative pain after SIES TEP might be comparable with conventional TEP. (LoE2)
4. Cosmetic scores might be better with SIES TEP compared to conventional TEP. (LoE3)
5. The recurrence rate might be comparable between SIES TEP and conventional TEP. (LoE4)
6. SIES TEP procedure might have higher costs than conventional TEP. (LoE4)

Single-incision hernia repair was developed with the intention to improve cosmetic outcomes and to lessen postoperative pain by reduced number of trocars, when compared to common multi-trocar procedure. Despite the potential benefits, until now this procedure has not gained much acceptance.

Selection of the papers from the literature included studies on primary, both unilateral and bilateral inguinal hernia in an elective setting, performed in a totally extraperitoneal (TEP) fashion. TEP was the most commonly performed procedure for hernia repair with a single-incision technique. Very limited information only could be found on the transabdominal preperitoneal (TAPP) approach. The literature search identified four RCTs [144–147] (n = 398) on this topic and one meta-analysis [148]. Only three of the four RCTs were included in this meta-analysis as one of the trials was published later. Moreover, many studies that were included in this meta-analysis did not pass our selection criteria.

All four RCTs compared SIES versus conventional laparoscopic TEP. All trials included 99 or 100 patients. In two trials, a longer operative time was reported for SIES TEP, this was statistically significant in one trial only. Pain scores were significantly better after SIES TEP in two trials, resp. 2 h, 1 day and 7 days postoperatively. One trial reported significantly better cosmetic scores at 6-week follow-up. No serious complications occurred. No recurrence was reported, even in the follow-up period up to 21 months. Authors of one study mention additional costs of US $340 per patient for the single-port repair when original Triport™ was used.

Several other prospective or retrospective studies reporting on single-incision inguinal hernia repair have been identified [149–156]. These studies were viewed for complications. Remarkably, one of the case series reported on an exceptionally high incidence of recurrent hernia (31/200) and port-site incisional hernia (15/200). A single surgeon performed all TEPs in this study. No deaths or serious morbidity was reported in any of the studies.

To summarize, based on current data it seems that SIES TEP is safe and feasible, although the benefits for the patient might be disputable. Disadvantages might be extra costs and a prolonged learning curve.

##### Recommendations

1. SIES inguinal hernia repair (TEP) could be offered to patients who are concerned about cosmesis as a safe and feasible approach when performed by experienced surgeons.

Grade of recommendation: Weak.

#### Ventral hernia

##### Statements

1. SIES ventral hernia repair might be feasible and safe in comparison with laparoscopic ventral hernia repair (LVHR). (LoE3)
2. Operative time in SIES ventral hernia repair might be comparable to LVHR. (LoE3)
3. Overall recurrence rate after SIES ventral hernia repair might be comparable to LVHR. (LoE3)

The rationale for the development of single-incision laparoscopic ventral herniorrhaphy was to decrease incisional hernia sites in patients prone to develop ventral hernias, to reduce wound complications and to improve cosmesis.

No randomized controlled trials comparing SIES ventral hernia repair with laparoscopic ventral hernia repair (LVHR) have been identified. Only one comparative study was found [157] and two prospective series reporting on 50 or more patients [158, 159].

In these studies, SIES ventral hernia repair was described in various clinical settings, including postoperative ventral hernias in obese patients with advanced age. Adhesiolysis was also feasible using this approach. In the comparative study, 15 SIES and 18 LVHR procedures were performed. The adhesion grade according to Zühlke was noted at surgery, and this was comparable between the procedures. The mean operative time was 78,2 ± 31,2 min for SIES and 73,5 ± 25,4 min for LVHR. After SIES, two seromas and one hematoma were reported, whereas two small bowel injuries and two seromas occurred with LVHR. One recurrent hernia was reported after SIES within a follow-up of up to 31 months. No recurrence occurred in the LVHR group within a follow-up of up to 42 months. No port-site hernias developed in either group. In the prospective series, no serious complications occurred, except for a bladder perforation; no port-site hernias were reported within a follow-up of 28 to 34 months.

##### Recommendations

1. Experienced surgeons could offer SIES ventral hernia repair to patients since it is considered feasible and seems as safe as conventional laparoscopic ventral hernia repair.

Grade of recommendation: Weak.

### New developments

#### Intragastric surgery

##### Statements

In selected patients (tumor location near esophagogastric junction or pre-pyloric):

1. SIES intragastric surgery might be feasible for the resection of submucosal stromal tumors. (LoE5)

A new development for gastric resection, an intragastric approach using a single-port device might have some advantages compared to conventional laparoscopy with multiple ports. However, no comparative studies on this topic have been found in the literature. Three case series [160–162] and one case report [163] could be identified, with a total of 32 patients. In all studies, the indications for surgery were submucosal stromal tumors, although the preoperative diagnosis was not always possible. There was heterogeneity with regard to the operating method, the type of devices, placement on the abdominal wall (umbilicus or left flank), the method for resecting the tumor (stapler or energy devices) and the use of intraoperative gastroscopy assisting the procedure. The devices used in the different series were a glove trocar with an Alexis wound retractor, the OCTO™ port and TriPortPlus™. The follow-up varied from 3 to 20 months, whereas the largest study (n = 21) reported on a follow-up of 19 months. The tumor locations were cardia, fundus and pylorus. In two studies with the largest cohorts, the mean tumor size was 2,4 ± 0,7 cm (n = 21) and 2,7 cm, range 2,3–3,8 (n = 7). The mean BMI was 22,6 ± 2,0 kg/m^2^ (n = 21) and 26,28 kg/m^2^, range 21–35 (n = 7). The mean operating time was 68,6 ± 12 min (n = 21) and 83,6 min, range 70–105 (n = 7). The mean hospital stay was 4,9 ± 1,7 days (n = 21) and 5,4 days, range 4–6 (n = 7).

The authors of the selected papers reported on the single-incision intragastric resection for the submucosal tumors as being feasible and with an acceptable number of complications. No mortality or major complications occurred. Surgeons did not experience any technical restrictions compared to their experience with conventional laparoscopic surgery. However, there are no randomized trials published yet with a head to head comparison of the single-port approach versus conventional laparoscopic approach to show and weigh the benefits and potential disadvantages.

##### Recommendations

No recommendation.

#### Single-port surgery through natural orifice

##### Statements

1. Natural orifice single access transvaginal cholecystectomy might have the potential to be safe and feasible and might represent a further advantage in terms of cosmesis and pain perception, compared to SIES. (LoE4)

Natural Orifice Transluminal Endoscopic Surgery (NOTES) is linked to the concept of surgery where access to the operative field is gained through natural orifices including the mouth, the anus and the vagina, without skin incisions. By definition, it is both technically and technologically demanding and surgeons all around the world have been exploring the possible applications for this technique and its limitations with caution.

Six eligible studies investigating single access NOTES have been identified [164–169]. The total population consists of 249 patients. Three of the included studies are comparative studies, and one is a randomized trial [164]. The randomized trial (n = 40) compared NOTES to standard laparoscopic surgery. The two non-randomized studies (n = 131) compared standard laparoscopy, SIES and NOTES. The remaining three papers are case series reporting on preliminary results of NOTES (n = 78). The performed procedures were cholecystectomy (transvaginal, transumbilical, transgastric), appendectomy (transvaginal, transumbilical, transgastric) and total mesorectal excision. The comparative studies explored merely cholecystectomy.

Transvaginal cholecystectomy (TVC) was performed in total in 45 patients. No conversion occurred in TVC procedures, neither to laparoscopic nor to open surgery. The operative time was similar when compared to transumbilical laparoscopic cholecystectomy (TLC). No deaths occurred. One case of intra-abdominal abscess was reported in the TVC group. In the randomized trial (n = 40), the postoperative pain was significantly lower in the TVC group compared to the TLC group (p < 0.001). In another prospective comparative study (n = 51), similar result was found.

Evidence on NOTES was generally of low quality. The few cases of transgastric-cholecystectomy, NOTES-total mesorectal excision, transgastric-appendectomy and transvaginal-appendectomy have been described only as preliminary results in case series. Comparative studies focused on single access transvaginal cholecystectomy. In total, merely 45 TVCs have been reported. This technique might have the potential to become an alternative to standard laparoscopic or single-incision laparoscopic cholecystectomy, providing improved cosmesis and less postoperative pain. However, high level of evidence is still lacking.

##### Recommendations

No recommendations.

#### Single-port and robotics

##### Statements

In selected robotic surgery (elective cholecystectomy, compatible with the da Vinci Si Surgical System):

1. Robotic SIES might be as safe and effective as standard laparoscopy but might be associated with a longer operating time. (LoE2)
2. Robotic SIES might offer an advantage in terms of cosmesis but not in terms of pain perception, compared to standard laparoscopy. (LoE2)
3. The risk of incisional hernia for robotic SIES compared to standard laparoscopy might be higher. (LoE2)

SIES robotic cholecystectomy (SIRC) is a recently introduced technique that is gaining acceptance among surgeons. The possibility to tackle the technical challenges of single-incision access by using robotic technology seems to be of great interest. The literature search identified thirteen studies with a total of 1411 patients [170–182]. Two studies were randomized controlled trials [170, 171] (n = 136 and n = 60), and three were non-randomized comparative studies [172–174] (n = 790). The two RCTs and a large non-randomized study (n = 678) compared SIRC with multi-port laparoscopic cholecystectomy (MPLC). Two smaller studies (n = 112) compared SIRC with single-incision laparoscopic cholecystectomy (SILC). The remaining eight papers (n = 425) were case series. All studies reported on elective cholecystectomy.

The RCT that included 136 patients had a follow-up of 3 months. The patients in the SIRC group had superior cosmesis satisfaction and body image perception (p < 0.05). However, there was no difference in quality of life. Operative time was longer in the SIRC group compared to multiport laparoscopy (61 min versus 44 min, p < 0.001). There was no difference in complication rates. In the RCT that included 60 patients, the cosmetic score 1 month postoperatively was higher for SIRC (p < 0.001). There was no difference in pain. A wound infection was reported in two SIRC patients. In this trial, patients were approached again 15 months after the end of the study to assess the occurrence of incisional hernia. One patient in the SIRC group developed a hernia, none in the MPLC group.

The third study, which compared SIRC to MPLC (n = 678), was a large comparative study with a retrospective analysis of prospectively maintained data. The majority, of the patients (n = 415, 61%), underwent a SIRC procedure. A single surgeon did all surgeries in this study. The total operative time was comparable between the groups. Hospital stay was shorter in the SIRC group (1,9 vs. 2,4 days). There were more wound infections in the SIRC group (3,9% vs. 1,1%). The rate of incisional hernia was higher in the SIRC group compared to MPLC group (27 vs. 5).

Other studies included in the review reported a higher rate of conversion to open surgery, higher rate of wound infections and wound abscesses for SIRC when compared to MLCP or SILS. There were neither deaths nor major complications. In the prospective series, 5 bleedings and 1 bowel injury were reported after the SIRC.

Two comparative studies reported on costs [173, 174]. One study reported comparable costs of instruments ($1268 for SIRC and $1282 for SILC). Another study reported on a total cost for SIRC. In this study, cost refers to the expense incurred to the hospital. There was no difference in variable direct supply cost or variable direct labor cost. However, fixed direct cost was significantly higher in the robotic group ($3137 for SIRC and $384 for SILC). The total cost was found to be higher in the SIRC group ($8961 for SIRC and $5379 for SILC). This is, according to the authors due to higher fixed costs, higher cost of administrative personnel for the operating room, and an increased operative time.

The single-incision robotic cholecystectomy seems to be a safe and effective technique. One point of concern though is that in several studies a higher incidence of incisional hernias after SIRC was demonstrated. Another point of concern could be the high costs associated with SIRC.

##### Recommendations

No recommendations.

## Conclusions

After viewing and discussing all the given evidence, the members of the EAES panel have formulated the following conclusions:

1. In general, there is a lack of high level evidence and a lack of long-term follow-up in the field of single-incision endoscopic surgery.
2. The available evidence does not give a sufficient platform to formulate strong recommendations.
3. If surgeons are willing to start with SIES, it is important to take into account the selection criteria of patients used in the studies for the specific topics.
4. In single-incision surgery for advanced procedures, the use of an extra-trocar from the beginning should be considered SIES.
5. In selected patients, the single-incision approach seems to be safe and effective in terms of perioperative morbidity.
6. Satisfaction with cosmesis has been established to be the main advantage of the single-incision approach.
7. Less pain after single-incision approach compared to conventional laparoscopy seems to be considered an advantage, although it has not been consistently demonstrated across studies.
8. There is a lack of evidence on the occurrence of incisional hernia after SIES in the long-term. Available data do not allow definitive conclusions regarding wound-related morbidity.
9. In selected patients, the oncological surrogate outcomes seem similar to that of the conventional laparoscopic approach; however, there is a lack of long-term data.
10. Considering the increased direct costs (devices, instruments and operating time) of the SIES procedure and the prolonged learning curve, wider acceptance of the procedure should be supported only after demonstration of clear benefits.
